# Associations Between Psychiatric Comorbidities and Cardiovascular Readmissions in Heart Failure: A Nationwide Analysis With a Focus on Substance Use Disorder

**DOI:** 10.7759/cureus.86933

**Published:** 2025-06-28

**Authors:** Kyle E Thurmann, Trisha G Mukherjee, Joseph G Dantin, Paul T Kang, Michael D White

**Affiliations:** 1 School of Medicine, Creighton University School of Medicine, Phoenix, USA; 2 School of Medicine, Rocky Vista University College of Osteopathic Medicine, Parker, USA; 3 Epidemiology and Public Health, Creighton University School of Medicine, Phoenix, USA; 4 Cardiology, Valleywise Health, Phoenix, USA; 5 Cardiology, Creighton University School of Medicine, Phoenix, USA

**Keywords:** cardiovascular disease, heart failure, hospital readmission, psychiatric comorbidities, substance use disorder, transitional care

## Abstract

Background

Psychiatric comorbidities are known to influence cardiovascular (CV) outcomes, but their specific impact on CV-specific readmissions following heart failure (HF) hospitalization remains unclear. Most prior research has focused on all-cause 30-day readmissions, limiting diagnosis-specific insight and long-term assessment.

Methods

We conducted a retrospective cohort study using the Nationwide Readmissions Database from 2016 to 2022. Adults (≥18 years) hospitalized with a primary diagnosis of HF were included. The psychiatric comorbidities of depression, anxiety, bipolar disorder, schizophrenia, post-traumatic stress disorder, and substance use disorder (SUD) were identified using ICD-10 codes. CV-specific readmissions at 30 days and one year were identified using ICD-10 codes for hypertension, HF or pulmonary edema, acute myocardial infarction, arrhythmias or conduction disorders, stroke or transient ischemic attack, pulmonary circulation disorders, and venous thromboembolism. Associations were evaluated using adjusted Cox regression models.

Results

Among 31,886,859 weighted hospitalizations, 6.1% (N = 1,945,098) had 30-day and 12.8% (N = 4,081,518) had one-year CV-specific readmissions. SUD was the only psychiatric condition independently associated with a higher hazard of CV-specific readmission at both time points: HR = 1.03 (95% CI: 1.02-1.03), p < 0.001 at 30 days, and HR = 1.02 (95% CI: 1.02-1.03), p < 0.001 at one year. All other psychiatric conditions were independently associated with a lower hazard of CV-specific readmission.

Conclusion

SUD may be a distinct risk factor for CV-specific readmissions following HF hospitalization and could benefit from targeted intervention. These findings emphasize the importance of diagnosis-specific transitional care and support the integration of psychiatric screening into CV risk stratification. While causality cannot be inferred due to the observational design, these results underscore the need for prospective studies to clarify underlying mechanisms.

## Introduction

The relationship between psychiatric disorders and cardiovascular disease (CVD) is well established. Mental health conditions, including depression, anxiety, post-traumatic stress disorder (PTSD), bipolar disorder, schizophrenia, and substance use disorder (SUD), have all been associated with elevated cardiovascular (CV) morbidity and mortality. For example, depression and anxiety have been linked to adverse CV outcomes through mechanisms such as systemic inflammation, increased sympathetic tone, and reduced medication adherence [1,2]. A large population study involving over three million patients and 113 million controls found that individuals with major depressive disorder, bipolar disorder, or schizophrenia experienced significantly higher CVD incidence and CV mortality than those without psychiatric illness [3]. Similarly, individuals with SUD are nearly twice as likely to have prevalent CVD, particularly those with opioid, stimulant, or alcohol use disorders [4,5]. From 1999 to 2019, age-adjusted mortality among individuals with comorbid SUD and CVD more than doubled [6].

In parallel, the economic and clinical burden of heart failure (HF) continues to escalate. Hospital readmissions, especially those due to recurrent HF decompensations, are a major contributor to morbidity and healthcare costs. In 2012, the total cost of HF care in the United States was estimated at $30.7 billion, with projections rising to $69.8 billion by 2030 [7]. Hospitalizations comprise more than 60% of HF-related healthcare costs, with readmissions further compounding the burden [8,9]. Identifying modifiable risk factors for readmission remains critical to improving patient outcomes and reducing costs.

While previous studies have consistently demonstrated associations between psychiatric illness and increased 30-day all-cause readmissions following HF or myocardial infarction (MI), fewer have isolated CV-specific readmissions as a primary endpoint [10,11]. This distinction is essential, as all-cause readmissions encompass a wide range of non-cardiac conditions, reducing diagnostic specificity and limiting the utility of targeted interventions. Additionally, the concept of post-hospital syndrome, a period of physiological vulnerability caused by hospital-associated stressors such as sleep deprivation, immobility, and nutritional deficits, may further exacerbate psychiatric instability and negatively impact CV outcomes in these patients [12].

To address this gap, we conducted a national analysis using the Nationwide Readmissions Database (NRD) to evaluate the impact of specific psychiatric comorbidities on 30-day and one-year CV-specific readmissions following HF hospitalization. We focused on six psychiatric conditions: depression, anxiety, bipolar disorder, schizophrenia, PTSD, and SUD. The objective of this study was to determine whether these disorders contribute differentially to CV-specific rehospitalization risk and whether such associations persist over the long term.

## Materials and methods

Data source

We performed a retrospective cohort study using the NRD, part of the Healthcare Cost and Utilization Project (HCUP), developed through a federal-state-industry partnership sponsored by the Agency for Healthcare Research and Quality. For this analysis, we used NRD data from 2016 to 2022, which includes discharge information from 30 states and represents approximately 60% of all U.S. hospitalizations and 61.1% of the U.S. resident population [13].

Study population and inclusion criteria

We included adult patients aged 18 years or older who were hospitalized with a primary diagnosis of HF, identified using ICD-10-CM codes I50.1 through I50.9. To ensure a consistent analytic cohort, we excluded patients who died during the index hospitalization, were discharged against medical advice, were transferred to another acute care facility, or had a planned readmission. Records missing essential demographic or outcome data were also excluded.

Psychiatric comorbidity definitions

To assess the effects of psychiatric comorbidities, we identified patients with one or more psychiatric diagnoses using the following ICD-10 codes: depression (F32.0-F32.9, F33.0-F33.9), anxiety (F40.0-F41.9), bipolar disorder (F31.0-F31.9), schizophrenia/psychotic disorders (F20.0-F20.9, F25.0-F25.9), PTSD (F43.10-F43.12), and SUDs (F10-F19, including all subcategories).

Outcome measures

The primary outcome was CV-specific readmission within 30 days or one year of index discharge. CV-specific readmissions were identified using ICD-10 codes for HF and pulmonary edema (I50.1-I50.9, J81.0-J81.1), acute MI (I21.0-I21.9), arrhythmias and conduction disorders (I47.0-I49.9), stroke and transient ischemic attack (I60.0-I63.9, G45.9), hypertension and hypertensive crisis (I10-I16), pulmonary circulation disorders (I26.0-I27.9), and venous thromboembolism (I82.0-I82.9).

Covariates and patient characteristics

We extracted patient-level demographic variables including age, sex, income quartile (based on ZIP code), and primary payer. Hospital-level characteristics included location (urban vs. rural), teaching status, and bed size. Additional covariates included admission day (weekend vs. weekday), discharge disposition (home, skilled nursing facility, home health, or other institutional care), and comorbidity burden, measured using the Charlson Comorbidity Index (CCI), a validated tool for predicting mortality and healthcare utilization [14].

Statistical analysis

We used weighted chi-squared tests to compare categorical variables and weighted linear regression for continuous variables. National estimates were generated using discharge-level weights provided by HCUP. Multivariable Cox proportional hazards regression was used to assess associations between psychiatric comorbidities and CV-specific readmissions at 30 days and one year. Hazard ratios (HRs) with 95% confidence intervals (CIs) were reported, representing the relative hazard of CV-specific readmission for patients with each psychiatric condition compared to those without the condition. All models were adjusted for patient demographics, hospital characteristics, and CCI to account for comorbidity burden. Secondary analyses included diagnosis-specific comparisons across psychiatric subtypes. A two-sided p-value < 0.05 was considered statistically significant.

Software and compliance

All analyses were conducted using STATA version 18 (StataCorp; College Station, TX). In compliance with the NRD Data Use Agreement, no descriptive statistics or results are presented for categories with ≤10 hospitalizations.

## Results

A total of 31,886,859 weighted hospitalizations were included in the analysis. The mean age was 71.3 years (SE 0.004), and 48.9% (N = 15,592,674) were female. Most patients were insured by Medicare (75.7%, N = 24,138,352), fell into the lowest income quartile (32.8%, N = 10,458,890), and resided in urban areas (82.4%, N = 26,274,772). The majority of patients were admitted to large hospitals (53.8%, N = 17,155,130) and urban teaching institutions (69.8%, N = 22,257,028). Nearly 75% (N = 23,883,257) of the population had a CCI ≥ 3, indicating substantial disease burden. Common discharge destinations included home (45.6%, N = 14,540,408), home healthcare (25.8%, N = 8,226,810), and designated care centers (25.7%, N = 8,194,923). Psychiatric comorbidity rates included 14.6% with depression (N = 4,655,481), 0.29% with anxiety (N = 92,472), 2.1% with bipolar disorder (N = 669,624), 1.5% with schizophrenia/psychotic disorders (N = 478,303), 0.8% with PTSD (N = 255,095), and 19.2% with SUD (N = 6,122,277). These demographics and clinical characteristics are shown in Table 1.

**Table 1:** Baseline demographic, clinical, and psychiatric characteristics of the study population This table presents weighted baseline characteristics for 31,886,859 hospitalizations of adults with heart failure in the Nationwide Readmissions Database. Variables include demographic factors (e.g., age, sex, income), insurance status, admission and discharge characteristics, and prevalence of psychiatric comorbidities. Depression (14.6%, N = 4,655,481) and substance use disorder (19.2%, N = 6,122,277) were the most common psychiatric diagnoses. All values are expressed as weighted percentages with standard errors. Abbreviations: N = weighted frequency; SE = standard error.

Demographics (Weighted)	Overall (n = 31,886,859)	
	% (N)	SE
Age, years	Mean = 71.3	0.004
Sex, female	48.9 (15,592,674)	0.013
Income quartile		
1	32.8 (10,458,890)	0.011
2	27.7 (8,832,660)	0.011
3	22.9 (7,302,091)	0.011
4	16.7 (5,325,105)	0.009
Primary payer		
Medicare	75.7 (24,138,352)	0.011
Medicaid	10.1 (3,220,573)	0.007
Private	10.0 (3,188,686)	0.008
Self-pay	1.79 (570,775)	0.003
Other	2.4 (765,285)	0.004
Weekend admission	24.4 (7,780,394)	0.011
Patient location		
Urban	82.4 (26,274,772)	0.008
Rural	17.6 (5,612,087)	0.008
Hospital location		
Urban/non-teaching	20.4 (6,504,919)	0.002
Urban/Teaching	69.8 (22,257,028)	0.003
Rural	9.8 (3,124,912)	0.002
Hospital bed size		
Small	18.5 (5,899,069)	0.002
Medium	27.7 (8,832,660)	0.002
Large	53.8 (17,155,130)	0.003
Charlson Comorbidity Index		
0–1	7.3 (2,327,741)	0.007
2	17.8 (5,675,861)	0.009
>3	74.9 (23,883,257)	0.011
Disposition at discharge		
Home	45.6 (14,540,408)	0.012
Short-term facility	1.2 (382,642)	0.003
Designated center	25.7 (8,194,923)	0.011
Home healthcare	25.8 (8,226,810)	0.011
Against medical advice	1.7 (542,077)	0.003
Unknown	0.047 (14,987)	6.5e-6
Psychiatric covariates		
Depression	14.6 (4,655,481)	0.009
Anxiety	0.29 (92,472)	0.001
Bipolar disorder	2.1 (669,624)	0.004
Schizophrenia/psychotic disorders	1.5 (478,303)	0.003
Post-traumatic stress disorder	0.8 (255,095)	0.002
Substance use disorder	19.2 (6,122,277)	0.01

Stratified analysis showed that 6.1% (N = 1,945,098) of patients were readmitted for CV-specific reasons within 30 days. Compared to those not readmitted, these patients were younger (mean age: 69.7 vs. 71.4 years; p < 0.001), less often female (46.7%, N = 908,361 vs. 49.1%, N = 14,701,405; p < 0.001), and more frequently covered by Medicaid (14.1%, N = 274,259 vs. 9.9%, N = 2,964,234; p < 0.001). They were disproportionately represented in the lowest income quartile (36.1%, N = 702,180 vs. 32.6%, N = 9,761,014) and were more often admitted on weekends (24.8%, N = 482,384 vs. 24.3%, N = 7,275,848). They were also more frequently discharged to home healthcare (28.0%, N = 544,627 vs. 25.6%, N = 7,665,091) or to home (46.8%, N = 910,306 vs. 45.5%, N = 13,623,501), more commonly treated at urban teaching hospitals (70.5%, N = 1,371,294 vs. 69.8%, N = 20,899,349), and had a greater overall comorbidity burden, with a higher proportion having a CCI ≥3 (79.4%, N = 1,544,408 vs. 74.6%, N = 22,336,554) (all p < 0.001). Full stratified results are presented in Table 2.

**Table 2:** Demographic and clinical characteristics stratified by 30-day cardiovascular-specific readmission status This table presents weighted demographic and clinical characteristics of patients with and without cardiovascular-specific readmissions within 30 days following an index hospitalization for heart failure in the Nationwide Readmissions Database (N = 31,886,859). Variables include age, sex, income quartile, insurance type, admission day, hospital location and size, Charlson Comorbidity Index, and discharge disposition. Patients readmitted within 30 days tended to be younger, less often female, and had higher comorbidity burdens. Categorical variables were analyzed using weighted chi-squared tests, while continuous variables were compared using weighted linear regression. Abbreviations: SE = standard error; N = weighted frequency.

Demographics (Weighted)	Cardiovascular-Specific 30-Day Readmission				p-value
	% (N)	SE	% (N)	SE	
Readmitted (no/yes)	No: 93.9 (29,941,761)		Yes: 6.1 (1,945,098)		
Age, years	Mean = 71.4	0.004	Mean = 69.7	0.014	< 0.001
Sex, female	49.1 (14,701,405)	0.013	46.7 (908,361)	0.051	< 0.001
Income quartile					< 0.001
1	32.6 (9,761,014)	0.012	36.1 (702,180)	0.051	
2	27.8 (8,323,810)	0.012	27.2 (529,067)	0.046	
3	22.9 (6,856,663)	0.011	24.6 (478,494)	0.042	
4	16.7 (5,000,274)	0.009	15.1 (293,710)	0.035	
Primary payer					< 0.001
Medicare	75.9 (22,725,797)	0.011	73.6 (1,431,592)	0.045	
Medicaid	9.9 (2,964,234)	0.007	14.1 (274,259)	0.035	
Private	10.1 (3,024,118)	0.008	9.1 (177,004)	0.028	
Self-pay	1.8 (538,952)	0.004	1.9 (36,957)	0.014	
Other	2.4 (718,602)	0.004	2.2 (42,792)	0.015	
Weekend admission	24.3 (7,275,848)	0.011	24.8 (482,384)	0.045	< 0.001
Patient location					< 0.001
Urban	82.3 (24,642,069)	0.008	83.9 (1,631,937)	0.039	
Rural	17.7 (5,299,692)	0.008	16.1 (313,161)	0.039	
Hospital location					< 0.001
Urban/non-teaching	20.4 (6,108,119)	0.003	20.8 (404,580)	0.038	
Urban/teaching	69.8 (20,899,349)	0.004	70.5 (1,371,294)	0.045	
Rural	9.8 (2,934,293)	0.003	8.7 (169,224)	0.030	
Hospital bed size					< 0.001
Small	18.5 (5,539,226)	0.003	17.9 (348,173)	0.039	
Medium	27.7 (8,293,868)	0.004	27.9 (542,682)	0.045	
Large	53.8 (16,108,667)	0.004	54.1 (1,052,298)	0.050	
Charlson Comorbidity Index					< 0.001
0-1	7.4 (2,215,690)	0.007	5.6 (108,925)	0.023	
2	17.9 (5,359,575)	0.011	15.0 (291,765)	0.037	
>3	74.6 (22,336,554)	0.012	79.4 (1,544,408)	0.042	
Disposition at discharge					< 0.001
Home	45.5 (13,623,501)	0.013	46.8 (910,306)	0.051	
Short-term facility	1.2 (359,301)	0.003	1.4 (27,231)	0.012	
Designated center	23.1 (6,916,547)	0.012	19.9 (387,075)	0.041	
Home healthcare	25.6 (7,665,091)	0.011	28.0 (544,627)	0.046	
Against medical advice	1.5 (449,126)	0.003	3.9 (75,859)	0.019	
Unknown	0.05 (14,971)	6.99e-6	2e-4 (4)	1.34e-6	

Comparable demographic and clinical patterns were observed among the 12.8% (N = 4,081,518) of patients who experienced CV-specific readmission within one year. These patients were also younger (70.0 vs. 71.5 years), less often female (47.3%, N = 1,930,558 vs. 49.2%, N = 13,680,228), and more frequently insured by Medicaid (13.5%, N = 551,005 vs. 9.6%, N = 2,669,313) (all p < 0.001). They were more likely to be discharged home (49.5%, N = 2,020,351 vs. 45.0%, N = 12,512,403) or to home healthcare (27.0%, N = 1,102,010 vs. 25.6%, N = 7,118,167), more often received care at urban teaching hospitals (70.7%, N = 2,885,633 vs. 69.7%, N = 19,380,323), and had higher comorbidity burdens (CCI ≥3: 77.9%, N = 3,179,503 vs. 74.5%, N = 20,714,979) (all p < 0.001). Full stratified results are presented in Table 3.

**Table 3:** Demographic and clinical characteristics stratified by one-year cardiovascular-specific readmission status This table summarizes weighted baseline characteristics of patients stratified by one-year cardiovascular-specific readmission following heart failure hospitalization using the Nationwide Readmissions Database (N = 31,886,859). Included variables are age, sex, income quartile, insurance coverage, timing of admission, hospital characteristics, Charlson Comorbidity Index, and disposition at discharge. Compared to non-readmitted patients, those with one-year readmissions were younger, had higher Medicaid use, and more often received care at large urban teaching hospitals. All comparisons reflect weighted national estimates with statistical significance assessed via weighted chi-squared and linear regression analyses. Abbreviations: SE = standard error; N = weighted frequency.

Demographics (Weighted)	Cardiovascular-Specific One-Year Readmission				p-value
	% (N)	SE	% (N)	SE	
Readmitted (no/yes)	No: 87.2 (27,805,341)		Yes: 12.8 (4,081,518)		
Age, years (mean)	Mean = 71.5	0.004	Mean = 70.0	0.010	< 0.001
Sex, female	49.2 (13,680,228)	0.013	47.3 (1,930,558)	0.036	< 0.001
Income quartile					< 0.001
1	32.4 (9,008,930)	0.012	35.9 (1,465,265)	0.035	
2	27.8 (7,729,885)	0.012	27.1 (1,106,091)	0.032	
3	23.0 (6,395,228)	0.011	21.7 (885,689)	0.029	
4	16.8 (4,671,297)	0.009	15.3 (624,472)	0.025	
Primary payer					< 0.001
Medicare	76.0 (21,132,059)	0.011	73.7 (3,008,079)	0.031	
Medicaid	9.6 (2,669,313)	0.009	13.5 (551,005)	0.024	
Private	10.2 (2,836,145)	0.008	8.4 (342,848)	0.020	
Self-Pay	1.7 (472,691)	0.004	2.1 (85,712)	0.010	
Other	2.4 (667,328)	0.004	2.2 (89,793)	0.010	
Weekend admission	24.3 (6,756,698)	0.012	24.5 (999,972)	0.031	< 0.001
Patient location					< 0.001
Urban	82.2 (22,855,990)	0.008	84.2 (3,436,638)	0.027	
Rural	17.8 (4,949,351)	0.009	15.8 (644,880)	0.027	
Hospital location					< 0.001
Urban/non-teaching	20.4 (5,672,290)	0.004	20.6 (840,793)	0.025	
Urban/teaching	69.7 (19,380,323)	0.005	70.7 (2,885,633)	0.030	
Rural	9.9 (2,752,729)	0.004	8.8 (359,174)	0.021	
Hospital bed size					< 0.001
Small	18.5 (5,143,988)	0.004	18.1 (738,755)	0.026	
Medium	27.7 (7,702,079)	0.005	27.7 (1,130,580)	0.03	
Large	53.8 (14,959,273)	0.006	54.2 (2,212,183)	0.034	
Charlson Comorbidity Index					< 0.001
0-1	7.5 (2,085,401)	0.007	6.0 (244,891)	0.016	
2	18.0 (5,004,961)	0.011	16.1 (657,124)	0.026	
>3	74.5 (20,714,979)	0.012	77.9 (3,179,503)	0.029	
Disposition at discharge					< 0.001
Home	45.0 (12,512,403)	0.013	49.5 (2,020,351)	0.036	
Short-term facility	1.2 (333,664)	0.003	1.0 (40,815)	0.007	
Designated center	26.6 (7,396,221)	0.012	19.6 (799,978)	0.028	
Home healthcare	25.6 (7,118,167)	0.012	27.0 (1,102,010)	0.031	
Against medical advice	1.5 (417,080)	0.003	2.9 (118,364)	0.012	
Unknown	0.054 (15,015)	7.52e-6	9.1e-7 (0)	6.43e-6	

Among psychiatric subgroups, schizophrenia/psychotic disorders (7.1%, N = 2,263,967) and SUD (7.0%, N = 2,232,080) had the highest 30-day CV-specific readmission rates. Adjusted Cox regression showed that SUD was the only psychiatric condition independently associated with a higher hazard of 30-day CV-specific readmission (HR: 1.03; 95% CI: 1.02-1.03; p < 0.001). In contrast, all other psychiatric conditions were associated with reduced risk, including depression (HR: 0.85; 95% CI: 0.84-0.85; p < 0.001), bipolar disorder (HR: 0.82; 95% CI: 0.81-0.84; p < 0.001), PTSD (HR: 0.84; 95% CI: 0.82-0.86; p < 0.001), schizophrenia/psychotic disorders (HR: 0.90; 95% CI: 0.89-0.92; p < 0.001), and anxiety (HR: 0.94; 95% CI: 0.90-0.98; p = 0.002).

**Figure 1:**
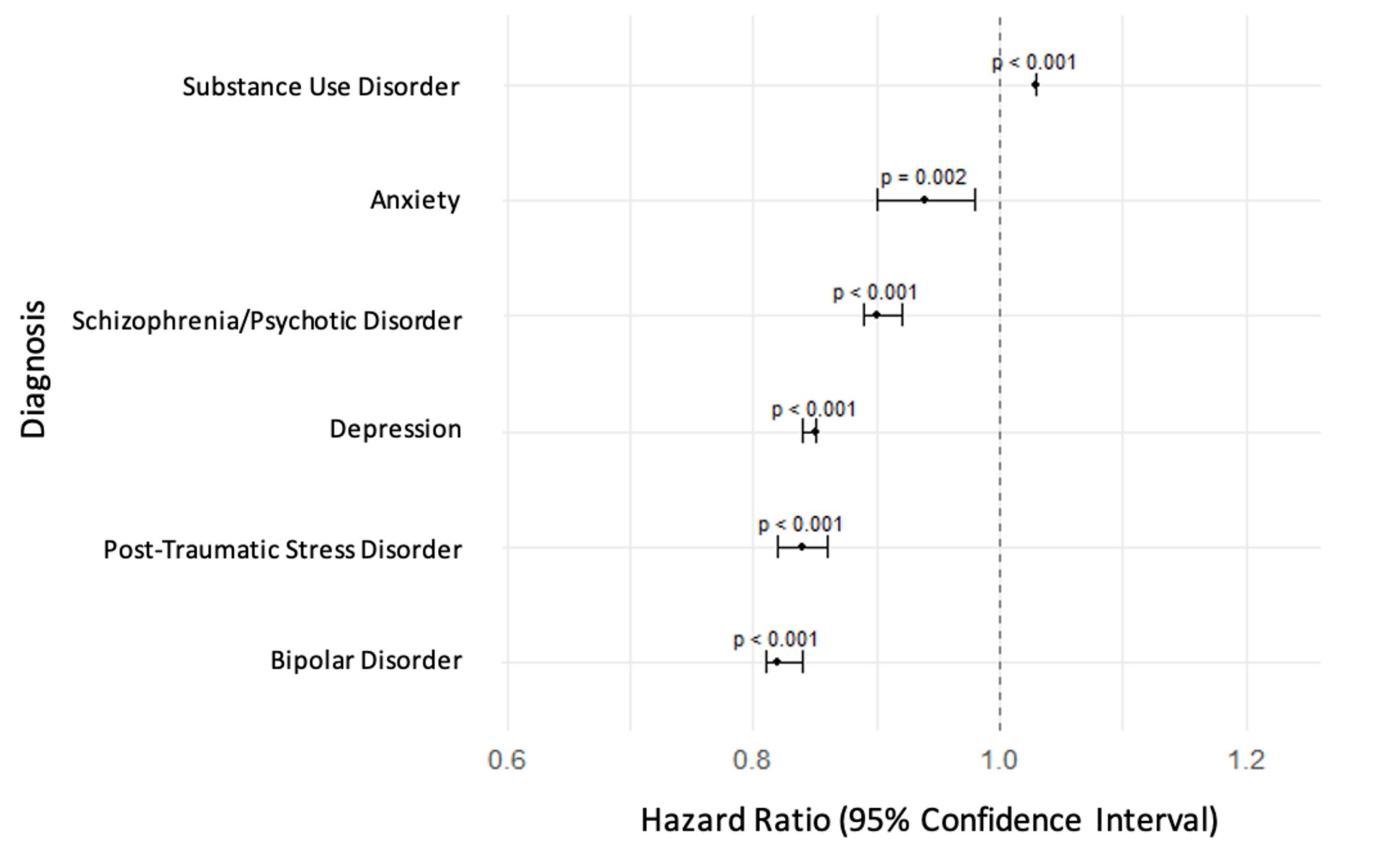
Adjusted hazard ratios for 30-day cardiovascular-specific readmission by psychiatric comorbidity This forest plot presents the multivariable-adjusted hazard ratios and 95% confidence intervals for 30-day cardiovascular-specific readmission following heart failure hospitalization, stratified by psychiatric comorbidity. All hazard ratios are derived from Cox proportional hazards models adjusted for demographics, socioeconomic status, and hospital characteristics. Substance use disorder was the only psychiatric diagnosis associated with a significantly increased hazard of cardiovascular-specific 30-day readmission (p < 0.001). All other psychiatric comorbidities, including depression, anxiety, bipolar disorder, schizophrenia/psychotic disorders, and post-traumatic stress disorder, were associated with significantly decreased hazards (all p < 0.01).

**Figure 2:**
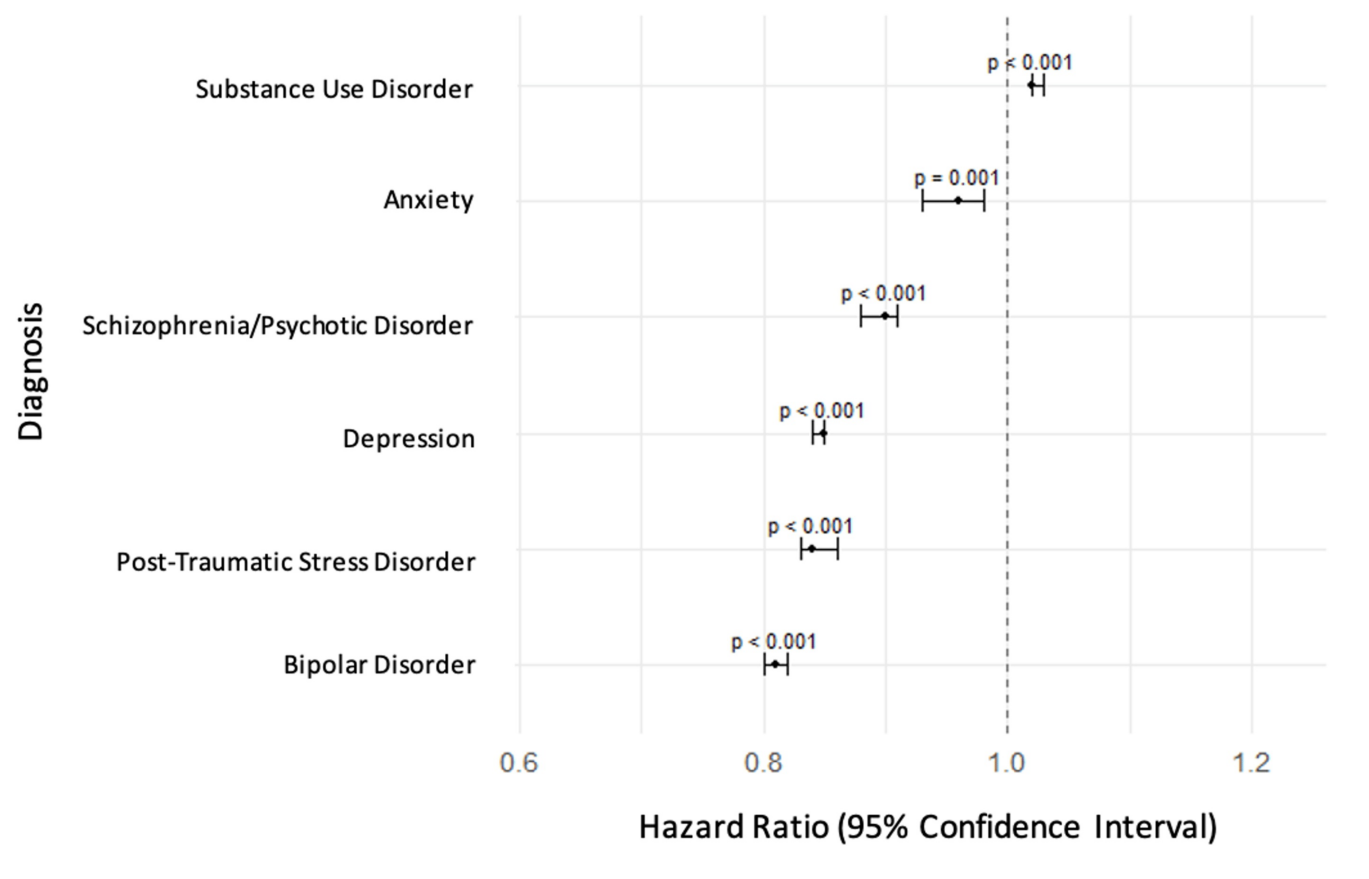
Adjusted hazard ratios for one-year cardiovascular-specific readmission by psychiatric comorbidity This forest plot presents the multivariable-adjusted hazard ratios and 95% confidence intervals for one-year cardiovascular-specific readmission following heart failure hospitalization, stratified by psychiatric comorbidity. All hazard ratios are derived from Cox proportional hazards models adjusted for demographics, socioeconomic status, and hospital characteristics. Substance use disorder was the only psychiatric diagnosis associated with a significantly increased hazard of cardiovascular-specific one-year readmission (p < 0.001). All other psychiatric comorbidities, including depression, anxiety, bipolar disorder, schizophrenia/psychotic disorders, and post-traumatic stress disorder, were associated with significantly decreased hazards (all p ≤ 0.001).

At one year, SUD (14.1%, N = 4,496,047) and schizophrenia/psychotic disorders (12.9%, N = 4,113,405) again had the highest CV-specific readmission rates. SUD remained the only psychiatric condition associated with an elevated hazard of readmission (HR: 1.02; 95% CI: 1.02-1.03; p < 0.001). All other psychiatric diagnoses were associated with reduced risk of one-year readmission: depression (HR: 0.85; 95% CI: 0.84-0.85; p < 0.001), bipolar disorder (HR: 0.81; 95% CI: 0.80-0.82; p < 0.001), PTSD (HR: 0.84; 95% CI: 0.83-0.86; p < 0.001), schizophrenia/psychotic disorders (HR: 0.90; 95% CI: 0.88-0.91; p < 0.001), and anxiety (HR: 0.96; 95% CI: 0.93-0.98; p = 0.001). 30-day and one-year results are presented in Table 4, with corresponding HRs illustrated in Figures 1, 2.

**Table 4:** Risk of cardiovascular-specific 30-day and one-year readmission by psychiatric comorbidities This table presents the weighted percentages and adjusted hazard ratios for 30-day and one-year cardiovascular-specific readmissions stratified by presence or absence of psychiatric conditions. Psychiatric comorbidities include depression, anxiety, bipolar disorder, schizophrenia/psychotic disorders, post-traumatic stress disorder, and substance use disorder. Multivariable Cox regression was used to estimate hazard ratios, adjusting for patient demographics, socioeconomic status, and hospital characteristics. All results are based on weighted estimates from the Nationwide Readmissions Database (N = 31,886,859). Substance use disorder was the only psychiatric condition associated with significantly increased hazard of cardiovascular-specific readmission at both time points. Abbreviations: SE = standard error; CI = confidence interval; HR = hazard ratio; REF = reference group; N = weighted frequency.

Psychiatric comorbidities	Cardiovascular-Specific 30-Day Readmission						Cardiovascular-Specific One-Year Readmission			
	% (N)		SE		HR (95% CI)	p-value	% (N)	SE	HR (95% CI)	p-value
Depression										
No	6.2 (1,976,985)		0.007		REF	< 0.001	12.9 (4,113,405)	0.009	REF	< 0.001
Yes	5.6 (1,785,664)		0.016		0.85 (0.84, 0.85)		11.6 (3,698,876)	0.022	0.85 (0.84, 0.85)	
Anxiety										
No	6.1 (1,945,098)		0.006		REF	0.002	12.7 (4,049,631)	0.009	REF	0.001
Yes	5.9 (1,881,325)		0.12		0.94 (0.90, 0.98)		12.3 (3,922,084)	0.16	0.96 (0.93, 0.98)	
Bipolar disorder										
No	6.1 (1,945,098)		0.006		REF	< 0.001	12.8 (4,081,518)	0.009	REF	< 0.001
Yes	6.3 (2,008,872)		0.044		0.82 (0.81, 0.84)		11.8 (3,762,649)	0.057	0.81 (0.80, 0.82)	
Schizophrenia/psychotic disorders										
No	6.1 (1,945,098)		0.006		REF	< 0.001	12.8 (4,081,518)	0.009	REF	< 0.001
Yes	7.1 (2,263,967)		0.053		0.90 (0.89, 0.92)		12.9 (4,113,405)	0.07	0.90 (0.88, 0.91)	
Post-traumatic stress disorder										
No	6.1 (1,945,098)		0.006		REF	< 0.001	12.8 (4,081,518)	0.009	REF	< 0.001
Yes	6.0 (1,913,212)		0.067		0.84 (0.82, 0.86)		11.8 (3,762,649)	0.091	0.84 (0.83, 0.86)	
Substance use disorder										
No	5.9 (1,881,325)		0.007		REF	< 0.001	12.4 (3,953,971)	0.009	REF	< 0.001
Yes	7.0 (2,232,080)		0.015		1.03 (1.02, 1.03)		14.1 (4,496,047)	0.02	1.02 (1.02, 1.03)	

**Figure 3:**
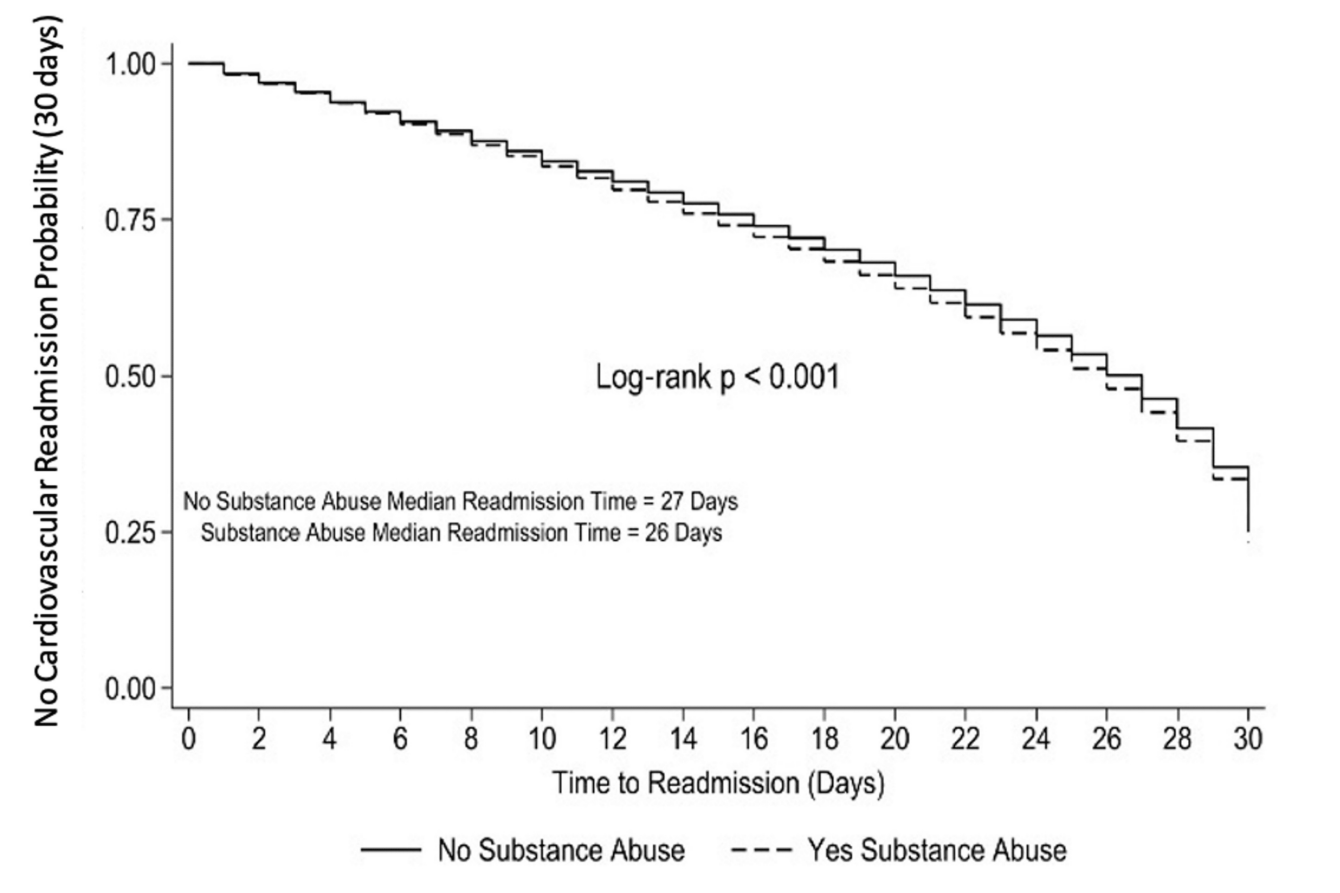
Kaplan-Meier curve for 30-day cardiovascular-specific readmission by substance use disorder status This Kaplan-Meier survival analysis compares time to cardiovascular-specific readmission within 30 days following heart failure hospitalization between patients with and without substance use disorder. Patients with substance use disorder experienced significantly earlier cardiovascular-specific readmissions than those without substance use disorder (log-rank p < 0.001). Median time to readmission was 26 days for the substance use disorder group and 27 days for the non-substance use disorder group.

**Figure 4:**
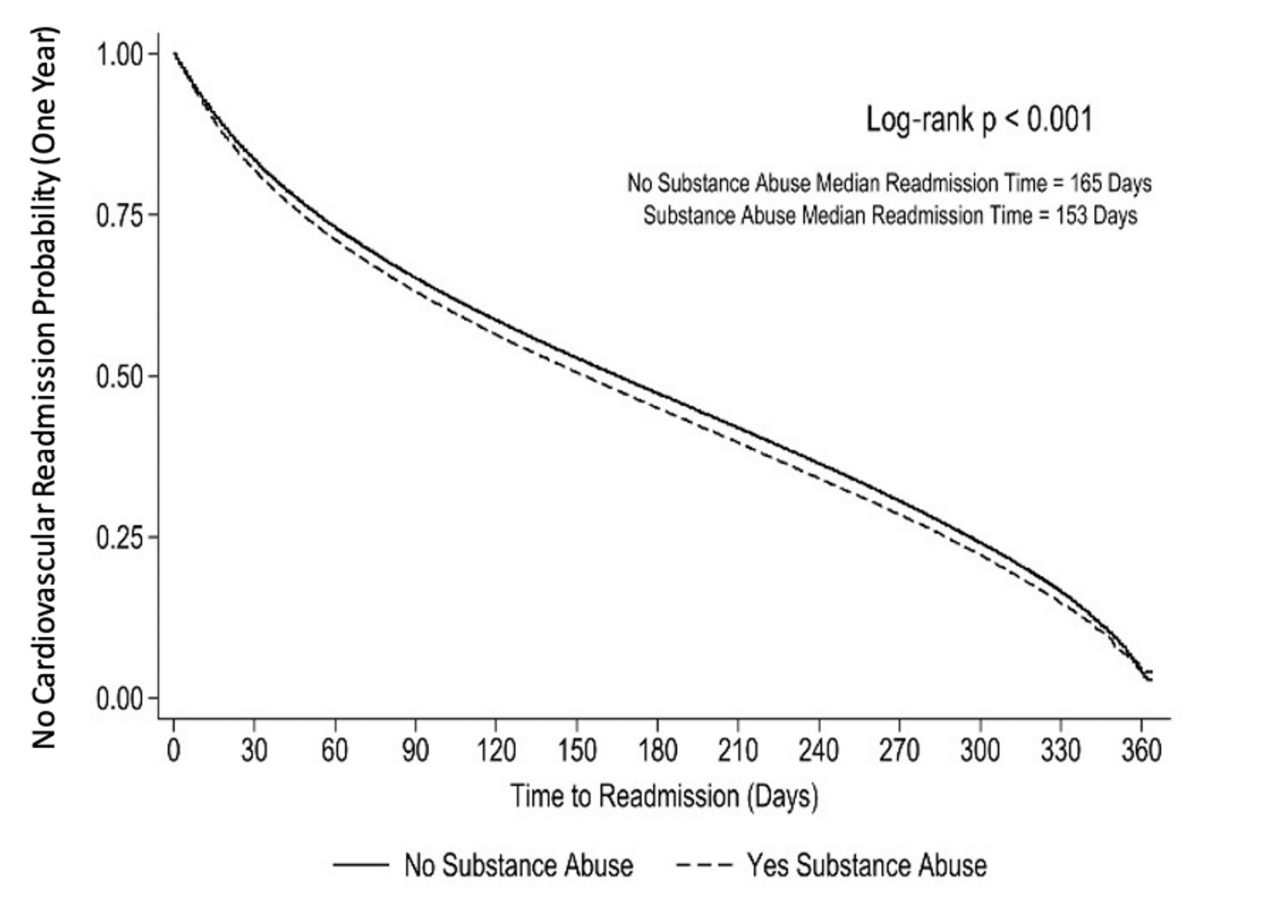
Kaplan-Meier curve for one-year cardiovascular-specific readmission by substance use disorder status This Kaplan-Meier survival analysis compares time to cardiovascular-specific readmission within one year following heart failure hospitalization between patients with and without substance use disorder. Patients with substance use disorder experienced significantly earlier cardiovascular-specific readmissions than those without substance use disorder (log-rank p < 0.001). Median time to readmission was 153 days for the substance use disorder group and 165 days for the non-substance use disorder group.

Kaplan-Meier survival curves demonstrated that patients with SUD had a significantly higher and earlier risk of CV-specific readmission compared to those without SUD at both 30 days and one year (Figures 3, 4; log-rank p < 0.001 for both). At 30 days, the SUD group showed a steeper decline in survival probability, with a median time to readmission of 26 days versus 27 days in the non-SUD group. At one year, the curves continued to diverge, indicating a persistently elevated cumulative risk of readmission among patients with SUD, with a median time to readmission of 153 days compared to 165 days in the non-SUD group.

## Discussion

This large-scale, nationally representative study examines how specific psychiatric conditions influence CV-specific readmissions following hospitalization for HF. While prior literature has consistently linked psychiatric illness to higher all-cause readmission rates, our findings diverge by demonstrating that most psychiatric comorbidities, including depression, bipolar disorder, PTSD, anxiety, and schizophrenia, were actually associated with lower CV-specific readmission risk. In contrast, SUD was the only psychiatric condition independently associated with an increased hazard of CV-specific readmission at both 30 days and one year. Additionally, Kaplan-Meier survival analyses showed that patients with SUD not only had higher overall readmission risk but also experienced earlier CV-specific rehospitalization events. Although the differences in median time to readmission were statistically significant, the absolute time differences between SUD and non-SUD groups were modest, suggesting limited clinical significance. This highlights the need for cautious interpretation, particularly due to the large sample.

This divergence highlights the value of diagnosis-specific metrics. All-cause readmission measures may obscure condition-specific risks and thereby misguide intervention efforts. Despite the well-known challenges faced by psychiatric patients, including fragmented care, socioeconomic adversity, and heightened medical burden, our results suggest that these factors may not necessarily translate into elevated CV risk. While causality cannot be inferred, the findings indicate that psychiatric illness alone may not drive CV-specific decompensations. Therefore, targeted behavioral, clinical, or social interventions might play a pivotal role in mitigating CV risk in certain psychiatric subgroups.

This work adds novel insights to existing literature by showing that SUD was uniquely associated with a higher hazard of both 30-day and one-year CV-specific readmissions, signaling a particularly high CV burden. For example, individuals with alcohol use disorder face a 1.5- to 2-fold higher risk of ischemic heart disease, emphasizing the impact of both the quantity and pattern of consumption [15]. A prospective cohort study of over 47,000 Japanese women revealed that heavy alcohol use (≥300 g/week) more than doubled the risk of both ischemic and hemorrhagic stroke [16].

Other substances, such as methamphetamine, opioids, cocaine, and cannabis, exert direct toxic effects on the myocardium, vasculature, and conduction system, contributing to arrhythmias, myocardial injury, hypertension, and cardiomyopathy [4,6]. Specifically, cocaine has been shown to induce sympathetic overactivation, coronary vasospasm, arrhythmias, and thrombosis, resulting in both acute and chronic myocardial injury [17]. Furthermore, large-scale electronic health record analyses have demonstrated elevated rates of arrhythmias, MI, and cerebrovascular events among regular users of cocaine and chronic cannabis, even after adjustment for confounders [18,19]. A scoping review of 23 studies provided consistent evidence linking chronic opioid use to increased risks of MI and infective endocarditis, with possible associations also observed for arrhythmias and stroke [20].

Additionally, a single-center study of over 11,000 patients with HF found that methamphetamine, opioid, and alcohol use were each independently associated with increased HF encounters, with incidence rate ratios comparable to classic comorbidities such as atrial fibrillation and ischemic heart disease [21]. Similarly, a national cohort of 184,000 HF patients showed that SUD, particularly stimulant use, was associated with significantly higher 30-, 180-day, and one-year readmission and mortality rates, even in a younger and less comorbid population [22]. Recent studies underscore these findings, highlighting alcohol and stimulant use, particularly methamphetamine, as rising contributors to HF in younger adults, with associated risks of arrhythmias, readmission, and mortality [23-25]. These patterns underscore the urgent need for focused research into the role of SUD in CV-specific hospital utilization.

Contributing to this elevated risk, patients with SUD frequently face barriers such as poor medication adherence, inconsistent follow-up, healthcare stigma, and systemic fragmentation, with these challenges being especially pronounced among younger individuals. A nationwide study of 18- to 39-year-olds hospitalized for CV events found that those with SUD had higher odds of in-hospital mortality and acute kidney injury, despite fewer traditional CV risk factors [26]. The disproportionate burden of conditions such as cardiac arrest, HF, atrial fibrillation, and MI in this demographic underscores the early and severe CV impact of substance use.

Our results support the development of enhanced transitional care strategies for HF patients with co-occurring SUD. Recommended strategies include early outpatient follow-up, addiction support services, integrated psychiatry-cardiology care models, and extended observation post-discharge. Given the chronic and relapsing nature of substance use, care pathways must also prioritize long-term monitoring and coordination.

We observed an inverse association between several other psychiatric conditions, including depression, anxiety, bipolar disorder, PTSD, and schizophrenia, and CV-specific readmission. Although initially counterintuitive, this may reflect increased clinical monitoring, psychosocial interventions, or use of case management in patients with established psychiatric diagnoses. Additionally, patients with severe psychiatric illnesses may be more frequently rehospitalized for non-CV reasons, which could reduce the observed rate of CV-specific readmissions in administrative datasets. 

Biological mechanisms may also play a role. For instance, depression is associated with autonomic dysfunction, inflammation, and platelet hyperreactivity, all of which may exacerbate CV outcomes [27]. Nevertheless, treatment with selective serotonin reuptake inhibitors and collaborative care models has shown promise in reducing depressive symptoms and may help improve prognosis. Similarly, PTSD has been independently linked to incident CVD and recurrent events, likely through pathways involving sympathetic overdrive and vascular dysfunction [28]. However, PTSD remains underdiagnosed in many CV populations, and its effect on HF readmissions may vary with healthcare engagement patterns.

Our sociodemographic analyses further illustrate the complexity of CV readmission risk. Patients with SUD-related readmissions were more likely to be younger, male, Medicaid-insured, discharged to non-home settings, and carry a higher comorbidity burden. These findings echo prior work linking social vulnerability and fragmented healthcare to increased readmission risk [8]. Therefore, post-acute transitions, especially among those discharged to skilled nursing or home health, represent a key window for targeted intervention. Still, these associations remain correlational and may not fully explain underlying disparities.

These findings should be interpreted in the context of several limitations. First, the NRD is an administrative dataset lacking clinical details such as ejection fraction, adherence, or psychosocial support. As such, our analysis is limited to the diagnoses and variables captured in discharge coding; detailed clinical histories, provider notes, or exclusions based on documentation quality were not available. Second, psychiatric diagnoses were identified using ICD-10 codes recorded in administrative claims data rather than standardized clinical assessments, introducing potential misclassification or variation in diagnostic criteria across institutions. The observed prevalence of anxiety was notably low, which may reflect coding limitations or underdiagnosis in administrative data. SUD may also be more readily recognized or coded than other psychiatric conditions, leading to differential representation. Third, the NRD does not enable patient tracking across multiple years, which may lead to underestimation of long-term trends. Fourth, unmeasured confounding, such as illness severity, social determinants, or substance use patterns, may influence our results. Fifth, CV-specific readmissions were defined using primary diagnosis codes, potentially omitting multifactorial causes. Lastly, because post-discharge mortality is not captured, the role of competing risks or survival bias could not be assessed. Nonetheless, the large sample size and condition-specific focus offer meaningful clinical and policy-relevant insights.

Future research should explore both pharmacologic and behavioral strategies aimed at reducing CV-specific rehospitalization in SUD populations. Prospective studies incorporating biomarkers along with clinical variables such as ejection fraction, medication adherence, and psychosocial factors may help overcome the limited clinical detail inherent to administrative datasets and clarify the mechanisms linking psychiatric comorbidities to CV-specific readmissions. Additionally, trials of integrated HF and addiction care models may inform evidence-based interventions for high-risk groups. Stratifying outcomes by specific CV readmission subtypes, such as MI and HF, may improve the precision of preventive care strategies.

## Conclusions

In this nationally representative study of over 31 million hospitalizations, we found that SUD was the only psychiatric condition independently associated with increased CV-specific readmissions at both 30 days and one year following hospitalization for HF. In contrast, other psychiatric conditions, including depression, anxiety, bipolar disorder, schizophrenia, and PTSD, were associated with a reduced risk of CV-specific readmission after adjustment. These findings challenge assumptions based on all-cause readmission data and emphasize the need for diagnosis-specific risk stratification. SUD, in particular, emerges as a high-priority target for post-discharge interventions aimed at reducing CV morbidity. Integrated CV and behavioral health care models may be essential for improving outcomes in this vulnerable, high-risk population.
